# Tailoring Red-to-Blue Emission in In_1−x_Ga_x_P/ZnSe/ZnS Quantum Dots Using a Novel [In(btsa)_2_Cl]_2_ Precursor and GaI_3_

**DOI:** 10.3390/molecules30010035

**Published:** 2024-12-26

**Authors:** Calem Duah, Ji-Seoung Jeong, Ji Yeon Ryu, Bo Keun Park, Young Kuk Lee, Seon Joo Lee

**Affiliations:** 1 Division of Advanced Materials, Korea Research Institute of Chemical Technology (KRICT), Daejeon 34114, Republic of Korea; calem@krict.re.kr (C.D.);; 2 Advanced Materials and Chemical Engineering, University of Science and Technology (UST), Daejeon 34113, Republic of Korea; 3 Department of Chemistry, Sungkyunkwan University (SKKU), Suwon 16419, Republic of Korea

**Keywords:** quantum dots, In_1−x_Ga_x_P, In precursor, red-to-blue emission, photoluminescence quantum yield

## Abstract

Ternary In_1−x_Ga_x_P quantum dots (QDs) have emerged as promising materials for efficient blue emission, owing to their tunable bandgap, high stability, and superior optoelectronic properties. However, most reported methods for Ga incorporation into the InP structure have predominantly relied on cation exchange in pre-grown InP QDs at elevated temperatures above 280 °C. This is largely due to the fact that, when heating In and P precursors in the presence of Ga, an InP/GaP core–shell structure readily forms. Herein, we introduce a novel synthesis approach using the indium precursor [In(btsa)_2_Cl]_2_ and GaI_3_ to fabricate In_1−x_Ga_x_P QDs in a single step at relatively low temperatures (200 °C). By adjusting the GaI_3_ content, we achieved controlled emission tuning from red to blue. Structural and compositional analysis through X-ray diffraction (XRD) and X-ray photoelectron spectroscopy (XPS) confirmed successful Ga^3+^ incorporation into the QD core, with a corresponding blue shift in the emission as GaI_3_ content increased. The synthesized QDs demonstrated a photoluminescence quantum yield (PLQY) of ~50% and a full width at half maximum (FWHM) of 45~62 nm, highlighting the potential of this synthesis method for advanced optoelectronic applications.

## 1. Introduction

Quantum dots (QDs), renowned for their size-dependent optical and electronic properties [1,2], have revolutionized fields such as electronics, photonics [3,4,5], and biotechnology [6,7]. However, concerns about their environmental impact and safety, particularly due to toxic elements like cadmium (Cd) [8,9,10], have spurred research into eco-friendly and biocompatible alternatives. As the demand for Cd-free quantum dots grows, indium phosphide (InP) QDs have emerged as a promising alternative, offering similar emission wavelengths, high efficiency, photostability, and solution processability to their Cd-based counterparts. The tunable emission spectra of InP QDs present immense potential for use in display [11,12,13] and lighting applications [14,15,16].

Despite significant advancements in synthesizing red- and green-emitting InP QDs, achieving blue-emitting InP variants remains a challenge due to the inherently small core sizes, often below 2 nm for green-emitting InP QDs [17], which complicates the synthesis and stability of blue-emitting InP. As a result, researchers have turned to alternative materials such as ternary In_1−x_Ga_x_P QDs [18] to overcome these limitations and achieve efficient blue emission. The incorporation of gallium into InP is anticipated to not only enable blue emission but also enhance absorption at 450 nm in blue-emitting quantum dots [19].

However, under the synthesis conditions of Ga-doped blue-emitting In_1−x_Ga_x_P QDs, it is challenging to suppress the formation of an InP/GaP core–shell [20,21] while promoting the formation of ternary In_1−x_Ga_x_P QDs [22,23,24]. Most reported syntheses of In_1−x_Ga_x_P have been achieved through cation exchange at elevated temperatures. Pietra et al. demonstrated Ga-for-Zn cation exchange in InZnP QDs, leading to the formation of a compositionally graded core–shell structure [24]. Similarly, Kim et al. reported Ga-for-In cation exchange at high temperatures and Ga^3+^ concentrations, achieving blue-emitting In_1−x_Ga_x_P/ZnSeS/ZnS QDs [18]. More recently, Gupta et al. synthesized In_1−x_Ga_x_P/ZnS core–shell QDs via molten salt cation exchange at high temperatures (280–300 °C) [22].

To achieve the synthesis of In_1−x_Ga_x_P in a single step at mild temperatures, selecting the appropriate precursor is critical due to the differences in reactivity stemming from the chemical hardness, ionic radii, and valency of Ga^3^⁺ compared to In^3+^ [25]. For example, gallium acetylacetonate (Ga(acac)_3_) tends to favor ternary In_1−x_Ga_x_P formation, while Ga oleate promotes InP/GaP core–shell growth [26]. Yoo et al. demonstrated that the reaction between indium myristate and gallium decanoate leads to the formation of InGaP alloyed QDs, while the reaction between indium myristate and gallium myristate results in the formation of InP/GaP core–shell QDs [27].

In this study, we successfully synthesized In_1−x_Ga_x_P QDs in a single step without cation exchange at relatively low temperatures (200 °C) by reacting the novel [In(btsa)_2_Cl]_2_ precursor and GaI_3_ with tris(dimethylamino)phosphine (P(DMA)_3_). We selected P(DMA)_3_ as it provides a safer and more cost-effective alternative compared to tris(trimethylsilyl)phosphine (P(TMS)_3_). Additionally, GaI_3_ was chosen due to its higher solubility in organic solvents and high reactivity with P(DMA)₃, enabling the efficient formation of In_1−x_Ga_x_P QDs. By carefully controlling the a mount of GaI_3_, we achieved In_1−x_Ga_x_P/ZnSe/ZnS core–shell QDs with tunable emission from red to blue. It is hypothesized that the bulkiness of the indium precursor slows its reactivity with P(DMA)_3_, preventing the premature formation of InP and favoring the formation of In_1−x_Ga_x_P. This work aims to provide a comprehensive understanding of the synthesis process and clarify the role of Ga^3^⁺ in tuning the emission wavelength.

## 2. Results and Discussion

**Figure 1:**
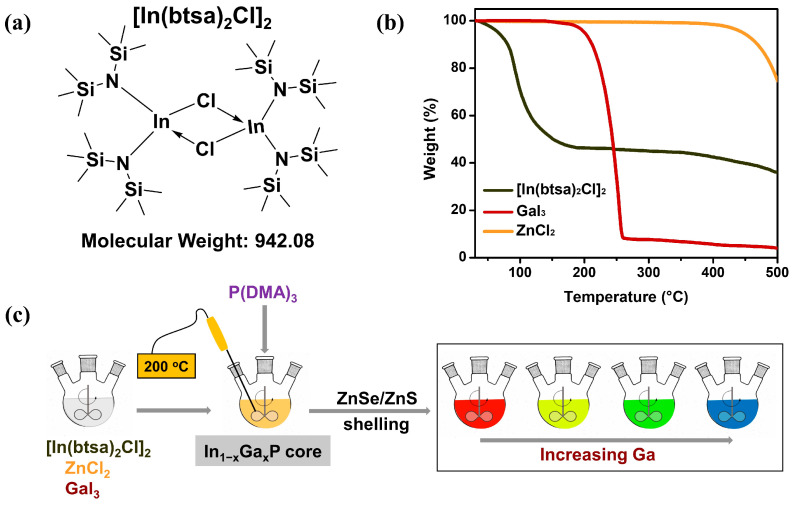
(**a**) Chemical structure of the [In(btsa)_2_Cl]_2_ precursor, (**b**) TGA curve of [In(btsa)_2_Cl]_2_, GaI_3_, and ZnCl_2_ measured over a temperature range of 30–500 °C at a heating rate of 10 °C/min, and (**c**) synthetic scheme for In_1−x_Ga_x_P/ZnSe/ZnS core–shell QDs.

In most studies reported to date, indium halides have been used as the precursors that react with tris(dimethylamino)phosphine (P(DMA)_3_) to form InP-based QDs. In this work, for the first time, we propose indium bis(bis(trimethylsilyl)amino)chloride, [In(btsa)_2_Cl]_2_ (Figure 1a), as a new precursor that reacts with P(DMA)_3_. [In(btsa)_2_Cl]_2_ was synthesized and purified in a glovebox using a modified method from the literature [28]. Thermogravimetric analysis (TGA) was conducted to investigate the evaporation characteristics of [In(btsa)_2_Cl]_2_, GaI_3_, and ZnCl_2_ as the temperature was gradually increased from 30 to 500 °C under a constant nitrogen flow, as shown in Figure 1b. The TGA trace of [In(btsa)_2_Cl]_2_ shows the three-step thermal decomposition of 9.2% (30–78 °C), 44.2% (78–188 °C), and 10.6% (188–500 °C). The final residue of [In(btsa)_2_Cl]_2_ was approximately 36% at 500 °C, and the TGA curve suggests continued mass loss beyond this point. The expected residue after the thermal decomposition of [In(btsa)_2_Cl]_2_ is either InN (27.3%) or In metal (24.4%). GaI_3_ exhibits a single-step weight loss in its TGA curve, leaving a residue of 9% after thermal evaporation in the temperature range of 165–258 °C. In contrast, ZnCl_2_ remains stable below 385 °C without premature volatilization. A previous study has reported that pure ZnCl_2_ begins to lose weight at approximately 400 °C, with the maximum weight loss observed at 587 °C [29].

The In_1−x_Ga_x_P-based QDs were synthesized following a modified procedure based on reference [19]. When [In(btsa)_2_Cl]_2_ was introduced, core formation occurred at a higher reaction temperature, above 200 °C, compared to using In halides. TGA results indicate that this temperature aligns with the decomposition temperature of a significant portion of the In precursor (Figure 1b). ZnCl_2_ was introduced at a molar ratio of 1:6.2 relative to the In precursor during the core formation process. Figure S1 shows that the bandgap change of the InP core upon the addition of ZnCl_2_ is negligible, whereas the addition of GaI_3_ induces a notable blue shift in the bandgap. These observations suggest that the majority of zinc remains on the surface of the QDs rather than within the core. It is well-known that zinc likely facilitates nucleation by regulating conversion kinetics and acting as a surface ligand on the In_1−x_Ga_x_P core, ultimately improving the photoluminescence quantum yield (PLQY) and narrowing the full width at half maximum (FWHM) [24,25]. To achieve monodisperse nanocrystals, the synthesized In_1−x_Ga_x_P cores were separated and purified [30], followed by the formation of a ZnSe inner shell and a ZnS outer shell, resulting in the production of In_1−x_Ga_x_P/ZnSe/ZnS core–shell QDs, as illustrated in Figure 1c. After the ZnS shell formation, a ligand treatment was carried out using 1-octanethiol (OTT) and zinc acetate dihydrate. OTT is well-known for its dual functionality as both a sulfur precursor and a surface stabilizer, facilitating effective surface passivation and enhanced photoluminescence properties of the QDs. A comparative analysis of In_1−x_Ga_x_P/ZnSe/ZnS QDs with and without ligand treatment revealed a significant improvement in PLQY, increasing from 24% (untreated) to 50% (treated), accompanied by a reduction in the FWHM from 75 nm to 62 nm (Figure S2). These results highlight the crucial role of ligand treatment in mitigating surface defects and enhancing monodispersity.

**Figure 2:**
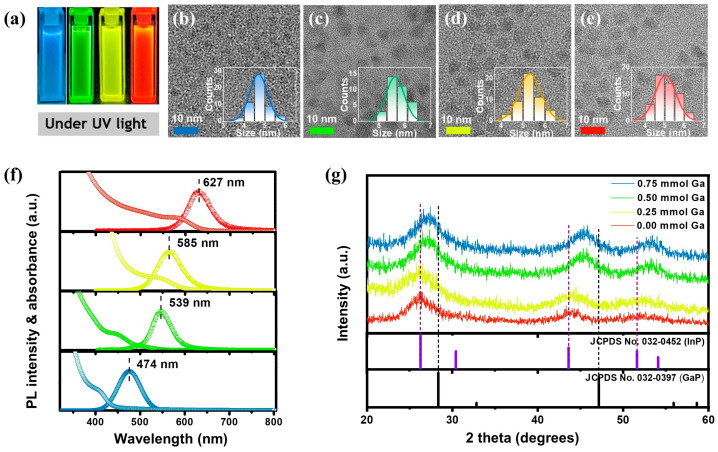
(**a**) Photographs of In_1−x_Ga_x_P/ZnSe/ZnS QD dispersions under UV irradiation. (**b**–**e**) TEM images and histograms of size distribution obtained by varying the amount of GaI_3_: (**b**) 0.75 mmol, (**c**) 0.50 mmol, (**d**) 0.25 mmol, and (**e**) 0.00 mmol during core synthesis. (**f**) Absorbance and photoluminescence spectra of In_1−x_Ga_x_P/ZnSe/ZnS QDs. (**g**) Powder X-ray diffraction patterns of In_1−x_Ga_x_P cores.

In_1−x_Ga_x_P-based QDs were successfully synthesized by introducing GaI_3_ in conjunction with [In(btsa)_2_Cl]_2_ during core formation. As the amount of GaI_3_ increased, more Ga^3+^ was incorporated into the In_1−x_Ga_x_P core, resulting in a gradual widening of the bandgap (Figure S3). This allowed for the production of In_1−x_Ga_x_P/ZnSe/ZnS core–shell QDs with tunable emission wavelengths ranging from red to blue (Figure 2a). Figure 2f shows the UV-Vis absorption and photoluminescence (PL) spectra of In_1−x_Ga_x_P/ZnSe/ZnS QDs with the stepwise addition of GaI_3_ in 0.25 mmol increments during core synthesis. With increasing amounts of GaI_3_, both the UV-Vis absorption and PL emission peaks gradually shifted toward shorter wavelengths. Initially, in the absence of GaI_3_, the PL peak was observed at 627 nm with an FWHM of 59 nm. Upon the addition of 0.25 mmol GaI_3_, the PL peak shifted to 585 nm with an FWHM of 62 nm, producing yellow-emitting QDs. Subsequent increases in GaI_3_ amount to 0.5 mmol and 0.75 mmol induced further blue shifts in the PL peak to 539 nm (FWHM: 50 nm) and 474 nm (FWHM: 45 nm), corresponding to green- and blue-emitting QDs, respectively. The PL peak wavelength (nm) and FWHM values (nm, cm^−1^) for each In_1−x_Ga_x_P/ZnSe/ZnS QD are listed in Table S1. The PLQY of In_1−x_Ga_x_P/ZnSe/ZnS core–shell QDs with increasing amounts of GaI_3_ was 32%, 50%, 31%, and 6%, respectively, with the highest PLQY observed for the yellow-emitting QDs at 0.25 mmol GaI_3_ (Supplementary Figures S4 and S5).

Figure 2b–e presents TEM images of blue-, green-, yellow-, and red-emitting In_1−x_Ga_x_P/ZnSe/ZnS QDs, respectively. Particle size measurements from TEM images of approximately 50 nanocrystals revealed that the blue-emitting QDs (0.75 mmol GaI_3_) have an average size of 4.67 ± 0.43 nm, the green-emitting QDs (0.50 mmol GaI_3_) measure 5.32 ± 0.35 nm, the yellow-emitting QDs (0.25 mmol GaI_3_) are 5.29 ± 0.51 nm, and the red-emitting QDs (without GaI_3_) measure 5.23 ± 0.50 nm. These results indicate that the particle size remains relatively constant when the amount of GaI_3_ is below 0.50 mmol but decreases when the amount of GaI_3_ exceeds 0.50 mmol. This implies that an excessive amount of GaI_3_ impedes the effective nucleation and growth of the In_1−x_Ga_x_P core. The comparable sizes of the green-, yellow-, and red-emitting QDs further suggest that the blue shift in emission is primarily due to the incorporation of Ga^3+^ rather than differences in particle size. This finding also serves as direct evidence for the formation of ternary In_1−x_Ga_x_P cores.

To further confirm the incorporation of Ga into the InP lattice, powder XRD analysis of the In_1−x_Ga_x_P core was conducted (Figure 2g). The XRD patterns of the In_1−x_Ga_x_P core are consistent with the cubic structure of bulk InP (JCPDS#032-0452) when the Ga content is zero (red-emitting QDs), with slight shifts toward the GaP bulk structure (JCPDS#032-0397) as the Ga content increases. The gradual shift toward larger angles with increasing GaI_3_ content, indicative of lattice contraction resulting from the substitution of indium with smaller gallium atoms in the crystal structure, confirms the formation of a ternary In_1−x_Ga_x_P core rather than an InP/GaP core–shell structure. Notably, minimal or no shifts in the diffraction patterns were observed as the GaI_3_ amount increased from 0.00 mmol to 0.25 mmol. However, a noticeable shift was observed when the GaI_3_ amount increased from 0.25 mmol to 0.50 mmol. The negligible shift in XRD patterns as the amount of GaI_3_ increases from 0.00 mmol to 0.25 mmol can be attributed to composition changes in the surface layer due to surface strain rather than bulk lattice reorganization [22]. Gupta et al. reported that, when a surface monolayer of InP is replaced with GaP, the XRD patterns may exhibit minimal changes, even though the elemental composition could contain up to 25% gallium. This suggests that Ga substitution at the surface alters the elemental composition of the QDs with little to no impact on the XRD patterns. Despite the possible presence of a Ga-substituted monolayer on the surface, there is no significant change in particle size (Figure 2d,e). Although the shift in XRD patterns from 0.50 mmol to 0.75 mmol of GaI_3_ remains subtle, TEM analysis confirms a reduction in particle size. Thus, the transition from green-emitting QDs to blue-emitting QDs is expected to be primarily due to the quantum confinement effect.

The formation mechanism of InP QDs from indium chloride (InCl_3_) and P(DMA)_3_ involves an initial adduct formation between the indium precursor (InCl_3_) and P(NHR)_3_, followed by nucleophilic attack by a second P(NHR)_3_ molecule, resulting in an InP intermediate and a P(NHR)_4_Cl phosphonium salt [31]. Generally, the rapid reaction between indium and phosphorus precursors in the presence of gallium precursor favors the formation of InP/GaP core–shell structures rather than ternary In_1−x_Ga_x_P QDs [26]. In this study, we employed a bulkier indium precursor, [In(btsa)_2_Cl]_2_, which introduces steric hindrance that slows the reaction kinetics. This slower reaction provides sufficient time for Ga incorporation, promoting the formation of ternary In_1−x_Ga_x_P QDs instead of InP/GaP core–shell structures.

**Figure 3:**
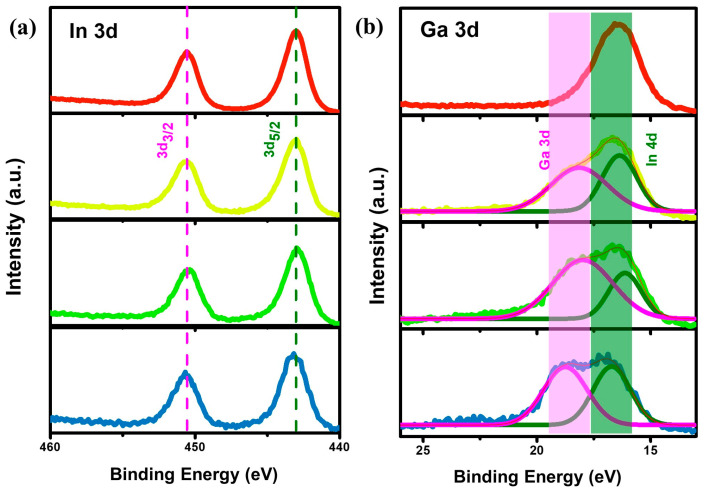
XPS high-resolution scans of (**a**) In 3d and (**b**) Ga 3d photoelectron peaks of In_1−x_Ga_x_P cores synthesized using 0.00 (red), 0.25 (yellow), 0.50 (green), and 0.75 (blue) mmol of GaI_3_.

To investigate the factors influencing wavelength shifts when nominal amounts of Ga were added, X-ray photoelectron spectroscopy (XPS) analysis was performed on red-, yellow-, green-, and blue-emitting In_1−x_Ga_x_P cores synthesized using the novel [In(btsa)_2_Cl]_2_ precursor (Figure 3). The Ga 3d photoelectron peaks, around 18 eV, consistently confirm the presence of gallium across the yellow-, green-, and blue-emitting In_1−x_Ga_x_P cores, as shown in Figure 3b. With increasing GaI_3_ content, the prominence of the Ga 3d shoulder peak intensifies, suggesting a greater likelihood of Ga^3+^ ions substituting In^3+^ ions within the core structure. When 0.25 mmol of GaI_3_ is introduced, the estimated gallium content is approximately 40%, despite no visible peak shift in the XRD pattern with the addition of 0.25 mmol Ga (red to yellow, Figure 2g). This is attributed to the surface substitution of Ga rather than bulk lattice reorganization, resulting in minimal changes in the XRD pattern. A Ga-rich surface toward the quantum dot surface explains the observed blue shift in absorbance. As the GaI_3_ content increases to 0.50 mmol, the gallium composition rises to 70.4%. However, beyond 0.50 mmol, the Ga composition decreases to approximately 53.8%, suggesting the system may have reached its maximum capacity for Ga incorporation.

**Figure 4:**
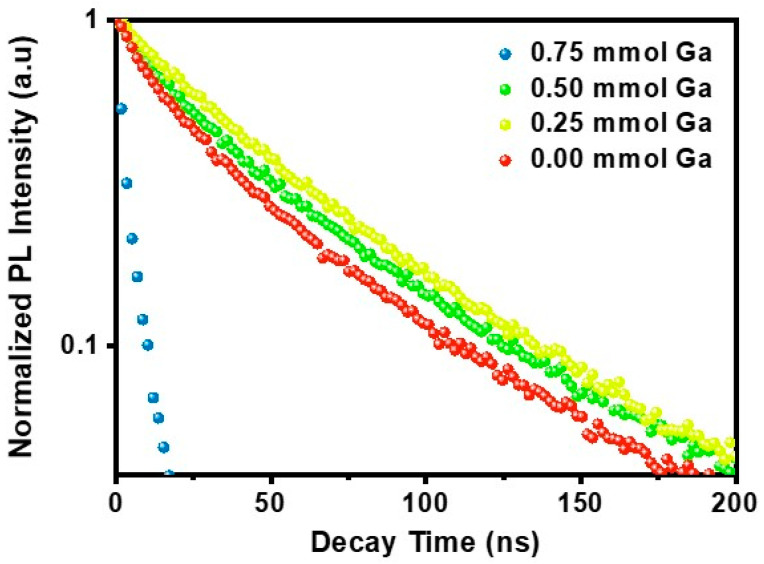
TRPL decay lifetime of In_1−x_Ga_x_P/ZnSe/ZnS QDs with different amounts of GaI_3_.

We performed time-resolved photoluminescence (TRPL) measurements on In_1−x_Ga_x_P/ZnSe/ZnS core–shell QD solutions and used bi-exponential fitting to analyze the fluorescence decay (Figure 4). The PL decay lifetimes for red-, yellow-, green-, and blue-emitting QDs are summarized in Table 1. For red-emitting QDs synthesized without GaI_3_, the average lifetime (*τ_avg_*) was 63.65 ns, increasing to 70.96 ns with the addition of 0.25 mmol GaI_3_ and to 70.87 ns with 0.50 mmol GaI_3_. The longer lifetime suggests enhanced radiative recombination efficiency attributed to moderate Ga incorporation, which likely passivates surface states and mitigates defects, as evidenced by an increase in the radiative decay rate constant (*k_r_*) and a decrease in the non-radiative decay rate constant (*k_nr_*). For QDs without GaI_3_, *k_r_* was measured at 4.71 µs⁻¹, while *k_nr_* was 10.99 µs⁻¹, indicating a predominance of non-radiative processes. Upon the addition of 0.25 mmol GaI_3_, *k_r_* increased to 7.05 µs⁻¹, and *k_nr_* decreased to 7.05 µs⁻¹, leading to a more balanced decay pathway favoring radiative recombination. However, further increasing GaI_3_ to 0.50 mmol caused *k_r_* to decrease to 4.23 µs⁻¹, accompanied by a reduction in PLQY to 31%. This decline suggests that, while initial Ga incorporation passivates defects and enhances radiative recombination, excessive Ga may introduce competing defect states, diminishing its beneficial effects [12,32]. A notable deviation is observed in the significantly shorter lifetime of blue-emitting In_1−x_Ga_x_P/ZnSe/ZnS QDs (6.12 ns). The significant reduction in carrier lifetime observed in blue-emitting QDs is primarily attributed to their decreased stability, which is a consequence of their high surface-to-volume ratio. TEM images in Figure 2b–e confirm that blue-emitting QDs have the smallest size compared to red-, yellow-, and green-emitting QDs. Furthermore, the blue-emitting QDs showed the poorest storage stability after synthesis, highlighting their intrinsic instability even after the shelling process. Therefore, this size-induced instability is considered the primary factor responsible for their unexpectedly short carrier lifetime. Additionally, a secondary factor may involve trap states generated by an excess of Ga or lattice strain effects, which facilitate non-radiative recombination pathways [22]. Furthermore, elevated Ga content may modify the surface composition, resulting in increased surface defects or poor passivation, effects that are particularly detrimental to smaller blue-emitting QDs. This interpretation is supported by a substantial increase in *k_nr_* to 153.4 µs⁻^1^, indicating that non-radiative processes account for approximately 94% of the total decay.

## 3. Materials and Methods

### 3.1. Materials

Indium(III) chloride(InCl_3_, 99.999%), potassium bis(trimethylsilyl)amide (95%), gallium iodide (GaI_3,_ 99.999%), zinc chloride (ZnCl_2_, 99.999%), selenium (100 mesh, 99.99%), sulfur (99.998%), oleylamine (OAm, C_18_H_35_NH_2_, 70%), oleic acid (OA, C_17_H_33_COOH, 90%), 1-octadecene (90%), zinc acetate dihydrate (Zn(ac)_2_.2H_2_O, 99.999%), trioctylphosphine (TOP, 90%), 1-octanethiol (OTT, 99%), zinc stearate (Tech. grade), and tris(dimethylamino)phosphine (P(DMA)_3_, 97%) were purchased from Sigma-Aldrich (Burlington, MA, USA). Tetrahydrofuran (THF), n-hexane and ethanol were purchased from Samchun Chemicals (Seoul, Republic of Korea). All chemicals were used without further purification.

### 3.2. Synthesis of Indium Bis(bis(trimethylsilyl)amino)chloride ([In(btsa)_2_Cl]_2_)

Indium bis(bis(trimethylsilyl)amino)chloride, [In(btsa)_2_Cl]_2_, was synthesized following a modified procedure based on existing literature [28]. In a glovebox, a solution of InCl_3_ (2.21 g, 0.01 mol) in THF (150 mL) was prepared, to which potassium bis(trimethylsilyl)amide (3.99 g, 0.02 mol) was added dropwise at room temperature. The reaction mixture was then stirred for 24 h under an argon atmosphere. Afterward, the volatile solvent was removed under reduced pressure, yielding the desired product as a white solid. This product was subsequently recrystallized from a saturated hexane solution at −20 °C, resulting in colorless crystals.

### 3.3. Synthesis of InP and In_1−x_Ga_x_P (x > 0) Core–Shell QDs

#### 3.3.1. Synthesis of InP and In_1−x_Ga_x_P (x > 0) Core

We synthesized InP quantum dots (QDs) using a novel indium complex, [In(btsa)_2_Cl]_2_ (Indium bis(bis(trimethylsilyl)amido)chloride), and P(DMA)_3_, following a modified version of previously reported procedures [19]. All reactions were carried out under a nitrogen atmosphere using standard Schlenk techniques. In a 100 mL three-necked flask, 0.175 g (0.186 mmol) of [In(btsa)_2_Cl]_2_, 0.157 g (1.15 mmol) of ZnCl_2_, and 3 mL of oleylamine were combined. The mixture was evacuated under vacuum at 120 °C for 60 min. The temperature was then raised to 200 °C, and a solution of 0.1 mL (0.55 mmol) P(DMA)_3_ mixed with 1 mL (2.24 mmol) tri-n-octylphosphine (TOP) was injected into the reaction mixture. The reaction was allowed to proceed for 20 min before being quenched to room temperature. The resulting mixture was washed with ethanol and hexane using centrifugation (10,000 rpm, 10 min) to remove impurities and excess reagents, and the final product was dispersed in 5 mL of hexane. For the synthesis of In_1−x_Ga_x_P cores, varying amounts of GaI_3_ (0.25, 0.5, 0.75 mmol) were added to the initial mixture of [In(btsa)_2_Cl]_2_ and ZnCl_2_.

#### 3.3.2. Growth of ZnSe Inner Shell

A mixture of 0.3475 g (0.55 mmol) zinc stearate, 3 mL of 1-octadecene, and 0.028 g (0.19 mmol) of the purified InP core dispersed in 4 mL hexane was loaded into a 100 mL three-necked flask. The mixture was evacuated under vacuum at 120 °C for 30 min, followed by raising the temperature to 160 °C. At this point, a Ga-treatment solution containing 0.024 g (0.053 mmol) of GaI_3_ in 1 mL of oleylamine (OAm) was injected into the flask. Immediately after this injection, a mixture of 0.120 g (0.19 mmol) zinc stearate in 2 mL of octadecene and a Se-TOP solution prepared from 0.01 g (0.125 mmol) Se in 1 mL (2.24 mmol) TOP were simultaneously injected into the reaction mixture. The reaction was maintained at 200 °C for 60 min to promote the growth of the ZnSe inner shell. Subsequently, the temperature was increased to 290 °C, and the reaction was allowed to proceed for an additional 30 min to complete the growth of the ZnSe shell.

#### 3.3.3. Growth of ZnS Outer Shell

For the growth of the ZnS outer shell, a mixture of 0.120 g (0.19 mmol) of zinc stearate in 2 mL of octadecene and a S-TOP solution were injected simultaneously into the reaction flask at 290 °C. The S-TOP solution was prepared by dissolving 0.016 g (0.5 mmol) of sulfur powder in 1 mL (2.24 mmol) of TOP at 120 °C. The reaction was allowed to proceed at 300 °C for 60 min to grow the ZnS outer shell.

#### 3.3.4. Ligand Treatment

The reaction temperature was lowered to 230 °C, and 0.2 mL of 1-octanethiol (OTT) was added dropwise and allowed to react for an additional 60 min. Subsequently, a solution containing 0.050 g (0.23 mmol) of zinc acetate dihydrate (Zn(ac)_2_.2H_2_O) dissolved in 1 mL of oleic acid (OA) was injected at 190 °C and allowed to react for 60 min. The reaction mixture was then cooled to room temperature. The same purification process used for the core synthesis was employed to collect the as-synthesized QDs, which were finally dispersed in 5 mL of hexane.

### 3.4. Characterization

The size and morphology of the QDs were assessed using high-resolution transmission electron microscopy (HRTEM, Spectra 300, ThermoFisher Scientific, Waltham, MA, USA) operated at an accelerating voltage of 200 kV. For TEM analysis, samples were prepared by depositing a few drops of diluted QD dispersion onto carbon-coated copper grids. The crystal structure of the QDs was investigated using X-ray diffraction (XRD, SmartLab, Rigaku, Tokyo, Japan) with operating conditions of 40 kV and 40 mA. The photoluminescence quantum yield (PLQY) was measured using a Quantaurus-QY Plus UV-NIR absolute PLQY spectrometer (C13534-11, Hamamatsu, Osaka, Japan). Photoluminescence (PL) spectra were recorded using a luminescence spectrometer (LS50B, PerkinElmer, Waltham, MA, USA) with an excitation wavelength of 365 nm. The absorption spectra of the QDs were obtained using a UV-Vis spectrophotometer (UV-2501PC, Shimadzu, Kyoto, Japan). Time-resolved photoluminescence (TRPL) spectra were characterized using a fluorescence lifetime spectrometer (Fluorolog-QM, Horiba, Kyoto, Japan). The composition and oxidation states of the QDs were analyzed by X-ray photoelectron spectroscopy (XPS, K-Alpha, ThermoScientific), equipped with a monochromatic Al Kα X-ray source (hv = 1486.6 eV). Thermogravimetric analysis (TGA) was performed using a thermogravimetric analyzer (TG209F1 Libra, Netzsch, Selb, Germany) with a nitrogen flow rate of 5 cc/min.

## 4. Conclusions

In this study, we successfully demonstrated the first synthesis of In_1−x_Ga_x_P/ZnSe/ZnS core–shell QDs using a novel indium precursor, [In(btsa)_2_Cl]_2_, and explored the effects of gallium incorporation on their structural and optical properties. The addition of GaI_3_ during core formation enabled tunable emission wavelengths ranging from red to blue, with a gradual blue shift as the Ga content increased. The highest photoluminescence quantum yield (PLQY) was observed for yellow-emitting QDs (0.25 mmol GaI_3_), highlighting enhanced radiative recombination efficiency in this system. This study underscores the potential of [In(btsa)_2_Cl]_2_ as an effective indium precursor for the synthesis of In_1−x_Ga_x_P/ZnSe/ZnS core–shell QDs with tunable optical properties, offering valuable insights into the influence of Ga incorporation on the emission behavior and structural characteristics of these QDs.

## Figures and Tables

**Table 1:** Kinetic fitting parameters obtained using bi-exponential fitting of TRPL traces with different GaI_3_ amounts in In_1−x_Ga_x_P/ZnSe/ZnS QDs: average lifetimes (*τ_avg_*), individual lifetimes (*τ*_1_ and *τ*_2_), corresponding amplitudes (A_1_ and A_2_), and calculated decay rate constants (total (*k_tot_*), radiative (*k_r_*), and non-radiative (*k_nr_*)), respectively.

GaI_3_ Amount (mmol)	A_1_	*τ*_1_ (ns)	A_2_	*τ*_2_ (ns)	*τ_avg_* (ns)	PLQY	*k_tot_* (µs^−1^)	*k_r_* (µs^−1^)	*k_nr_* (µs^−1^)
0.00	0.57	18.58	0.43	77.99	63.65	32%	15.7	4.71	10.99
0.25	0.65	35.67	0.35	95.20	70.96	50%	14.1	7.05	7.05
0.50	0.58	24.25	0.42	88.62	70.87	31%	14.1	4.23	9.87
0.75	0.71	1.85	0.32	8.24	6.12	6%	163.4	10	153.4

## Data Availability

All relevant data from this investigation are included in the article. Additional data supporting the findings of this study can be obtained from the corresponding authors upon reasonable request.

## Supplementary Materials

The following supporting information can be downloaded at: https://www.mdpi.com/article/10.3390/molecules30010035/s1, Table S1: PL peak wavelength (nm), FWHM (nm), and FWHM (10^5^ cm^−1^) with different GaI_3_ amount in In_1−x_Ga_x_P/ZnSe/ZnS QDs; Figure S1: UV-Vis absorption spectra of In_1−x_Ga_x_P core with varying ZnCl_2_ and GaI_3_ amounts; Figure S2: (a) Absorbance and (b) PL spectra of yellow-emitting In_1−x_Ga_x_P/ZnSe/ZnS QDs (Ga 0.50 mmol) with and without ligand treatment; Figure S3: Absorbance spectra of In_1−x_Ga_x_P cores synthesized using 0.00 (red), 0.25 (yellow), 0.50 (green), and 0.75 (blue) mmol of GaI_3_; Figure S4: PLQY spectra of In_1−x_Ga_x_P/ZnSe/ZnS QDs synthesized using (a) 0 (red), (b) 0.25 (yellow), (c) 0.5 (green), and (d) 0.75 (blue) mmol of GaI_3_; Figure S5: Magnified PLQY spectra of In_1−x_Ga_x_P/ZnSe/ZnS QDs synthesized using (a) 0 (red), (b) 0.25 (yellow), (c) 0.5 (green), and (d) 0.75 (blue) mmol of GaI_3_.
