# Supplementary material for: Tailoring Red-to-Blue Emission in In1−xGaxP/ZnSe/ZnS Quantum Dots Using a Novel [In(btsa)2Cl]2 Precursor and GaI3
Source: Molecules. 2024 Dec 26;30(1):35. doi: 10.3390/molecules30010035 (PMC11721170; doi:10.3390/molecules30010035)
Supplement: Supplementary file 1 [file molecules-30-00035-s001.zip › molecules-3286122-supplementary.pdf]

Supplementary Materials

Tailoring Red-to-Blue Emission in $In_{1-x}Ga_xP$/ZnSe/ZnS Quantum Dots using a Novel $[In(btsa)_2Cl]_2$ Precursor and GaI_3

Calem Duah [1,2], Ji-Seoung Jeong [1,3], Ji Yeon Ryu [1], Bo Keun Park [1,2], Young Kuk Lee [1] and Seon Joo Lee [1,2,*]

1 Division of Advanced Materials, Korea Research Institute of Chemical Technology (KRICT), 141 Gajeong-ro, Yuseong-gu, Daejeon 34114, Republic of Korea
2 Department of Advanced Materials and Chemical Engineering, University of Science and Technology (UST), 217 Gajeong-ro, Yuseong-gu, Daejeon 34113, Republic of Korea
3 Department of Chemistry, Sungkyunkwan University (SKKU), 2066 Seobu-ro, Jangan-gu, Suwon-si, Gyeonggi-do 16419, Republic of Korea
* Correspondence: sjlee614@krict.re.kr

Table S1. PL peak wavelength (nm), FWHM (nm), and FWHM (10^5 cm^{-1}) with different GaI_3 amount in $In_{1-x}Ga_xP$/ZnSe/ZnS QDs.

GaI_3 amount (mmol)	PL Peak (nm)	FWHM (nm)	FWHM (10^5 cm^{-1})
0.00	627	59	1.7
0.25	585	62	1.6
0.50	539	50	2.0
0.75	474	45	2.2

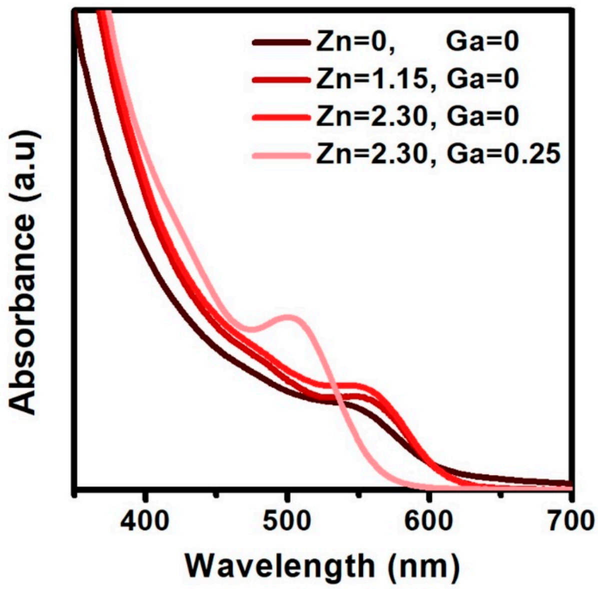

Figure S1. UV-Vis absorption spectra of $In_{1-x}Ga_xP$ core with varying $ZnCl_2$ and GaI_3 amounts.

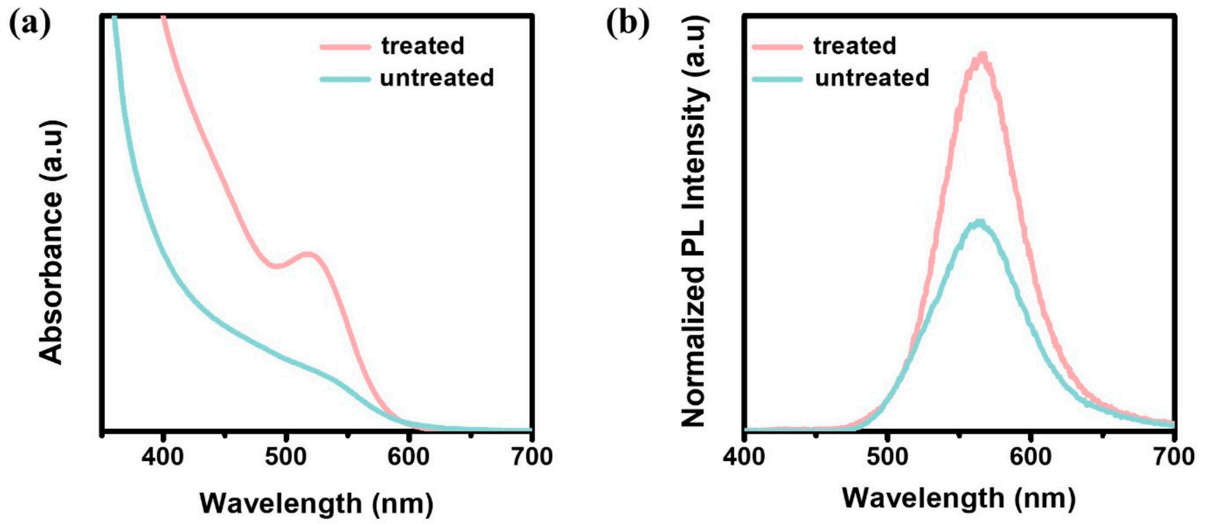

Figure S2. (a) Absorbance and (b) PL spectra of yellow-emitting $In_{1-x}Ga_xP/ZnSe/ZnS$ QDs (Ga 0.50 mmol) with and without ligand treatment.

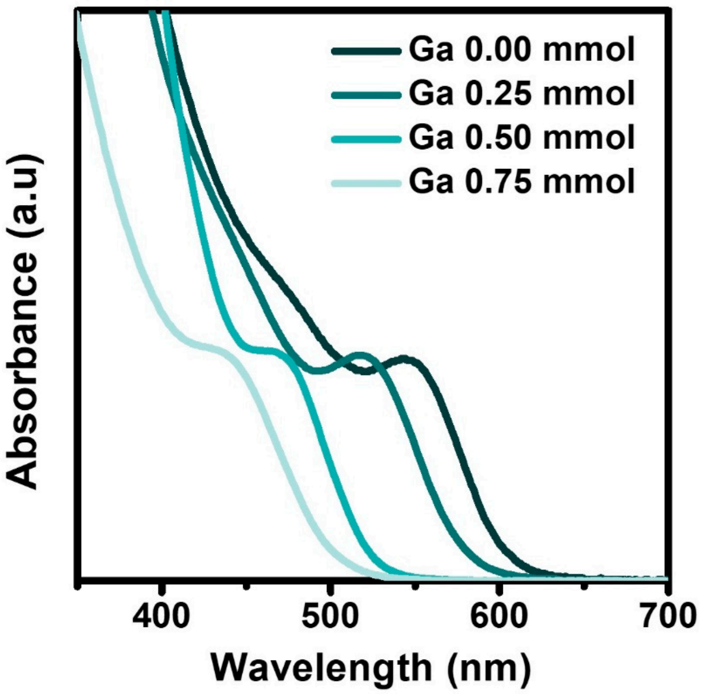

Figure S3. Absorbance spectra of $In_{1-x}Ga_xP$ cores synthesized using 0.00 (red), 0.25 (yellow), 0.50 (green), and 0.75 (blue) mmol of GaI_3.

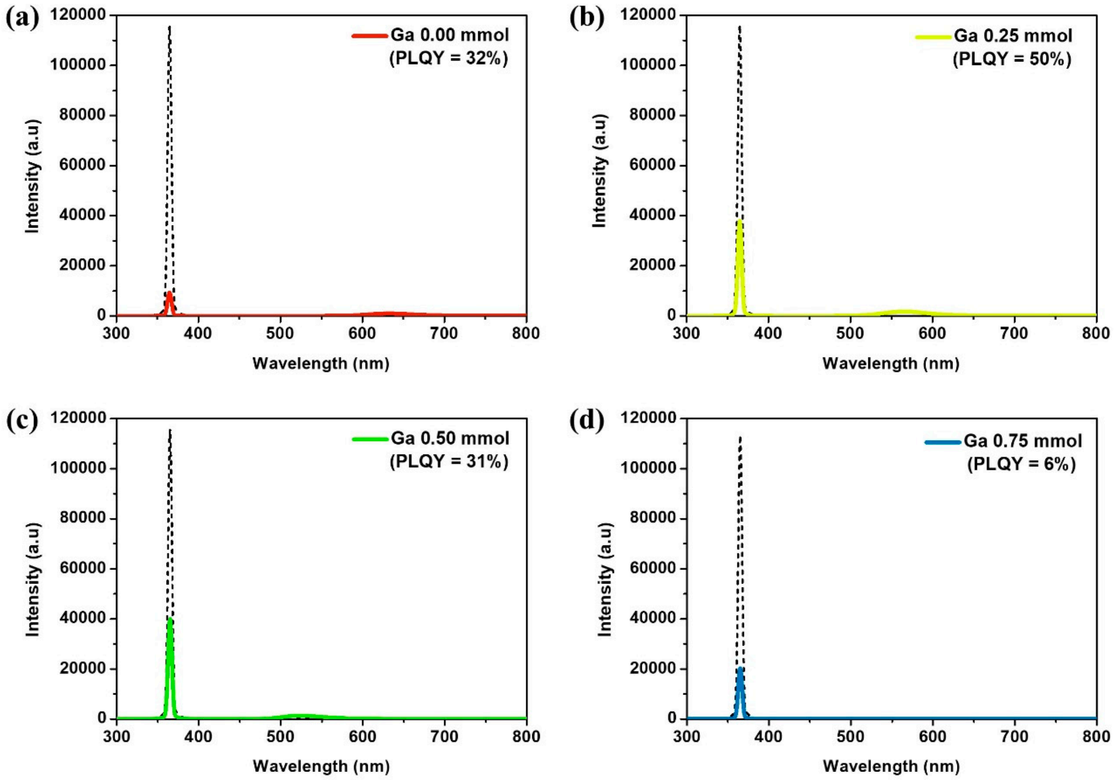

Figure S4. PLQY spectra of $In_{1-x}Ga_xP$/ZnSe/ZnS QDs synthesized using (a) 0 (red), (b) 0.25 (yellow), (c) 0.5 (green), and (d) 0.75 (blue) mmol of GaI_3

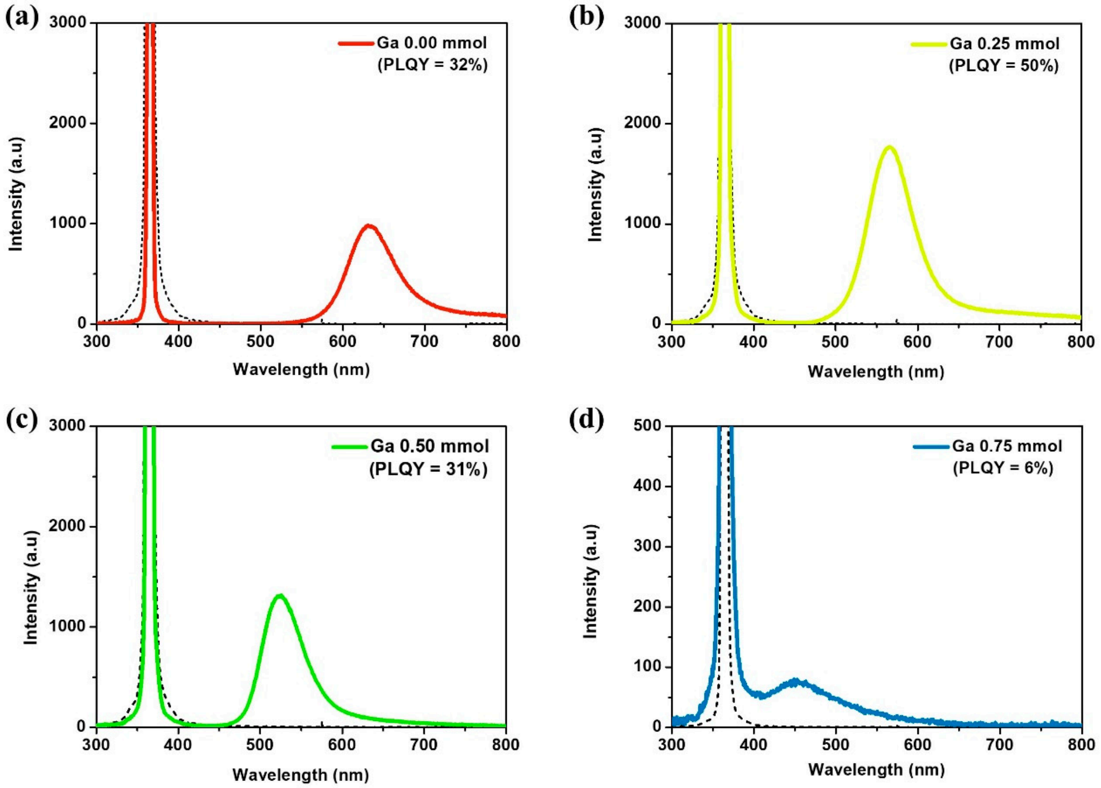

Figure S5. Magnified PLQY spectra of $In_{1-x}Ga_xP$/ZnSe/ZnS QDs synthesized using (a) 0 (red), (b) 0.25 (yellow), (c) 0.5 (green), and (d) 0.75 (blue) mmol of GaI_3
